# Chitinase 3-like 1 is induced by *Plasmodium falciparum* malaria and predicts outcome of cerebral malaria and severe malarial anaemia in a case–control study of African children

**DOI:** 10.1186/1475-2875-13-279

**Published:** 2014-07-21

**Authors:** Laura K Erdman, Carlene Petes, Ziyue Lu, Aggrey Dhabangi, Charles Musoke, Christine M Cserti-Gazdewich, Chun Geun Lee, Wayne Conrad Liles, Jack A Elias, Kevin C Kain

**Affiliations:** 1 S.A. Rotman Laboratories, Sandra Rotman Centre for Global Health, Toronto General Hospital-University Health Network, MaRS building Room 10-401, 101 College St, Toronto, Ontario M5G 1L7, Canada; 2 Department of Microbiology and Immunology, Queen’s University, Kingston, Ontario, Canada; 3 Makerere University College of Health Sciences, Kampala, Uganda; 4 Laboratory Medicine Program (Transfusion Medicine), University Health Network/University of Toronto, Toronto, Ontario, Canada; 5 Yale University School of Medicine, New Haven, Connecticut, USA; 6 Department of Medicine, University of Washington, Seattle, Washington, USA; 7 Brown University Biology and Medicine, Providence, Rhode Island, USA

**Keywords:** Cerebral malaria, Severe malaria, Chitinase 3-like 1, CHI3L1, Biomarker, Pathogenesis, Inflammation

## Abstract

**Background:**

Severe and fatal malaria are associated with dysregulated host inflammatory responses to infection. Chitinase 3-like 1 (CHI3L1) is a secreted glycoprotein implicated in regulating immune responses. Expression and function of CHI3L1 in malaria infection were investigated.

**Methods:**

Plasma levels of CHI3L1 were quantified in a case–control study of Ugandan children presenting with *Plasmodium falciparum* malaria. CHI3L1 levels were compared in children with uncomplicated malaria (UM; n = 53), severe malarial anaemia (SMA; n = 59) and cerebral malaria (CM; n = 44) using the Kruskall Wallis-test, and evaluated for utility in predicting fatal (n = 23) *versus* non-fatal (n = 80) outcomes in severe disease using the Mann Whitney U test, receiver operating characteristic curves, and combinatorial analysis. Co-culture of *P. falciparum* with human peripheral blood mononuclear cells and the *Plasmodium berghei* ANKA experimental model of cerebral malaria were used to examine the role of CHI3L1 in severe malaria.

**Results:**

In children presenting with falciparum malaria, CHI3L1 levels were increased in SMA and CM *versus* UM (p < 0.001). Among severe malaria cases, CHI3L1 levels at presentation predicted subsequent death (area under receiver operating characteristic curve 0.84 [95% CI 0.76-0.92]) and in combination with other host biomarkers, predicted mortality with high sensitivity (100% [85.7-100]) and specificity (81.3% [71.3-88.3]). *Plasmodium falciparum *stimulated CHI3L1 production by human peripheral blood mononuclear cells *in vitro*. CHI3L1 was increased in plasma and brain tissue in experimental cerebral malaria, but targeted *Chi3l1* deletion did not alter cytokine production or survival in this model.

**Conclusions:**

These data suggest that plasma CHI3L1 measured at presentation correlates with malaria severity and predicts outcome in paediatric SMA and CM, but do not support a causal role for CHI3L1 in cerebral malaria pathobiology in the model tested.

## Background

Malaria causes an estimated 450 million infections and 1.24 million deaths annually [1,2]. Most deaths are attributable to severe *Plasmodium falciparum* infection in African children. The two major severe malaria syndromes are cerebral malaria (CM), which presents as coma and/or seizures, and severe malarial anaemia (SMA). Case fatality rates for CM and SMA are 18% and 10%, respectively, despite optimal anti-malarial treatment [3]. An improved understanding of pathogenesis is required to develop adjunctive therapies, as well as prognostic tools to guide triage and appropriate allocation of limited health care resources.

While the sequestration of parasitized erythrocytes is central to severe malaria pathogenesis, disease progression is also influenced by host responses to infection, including inflammation. A robust T_H_1 response is critical for control of parasite replication, but severe disease is associated with dysregulated inflammation. Children with CM have elevated serum/plasma T_H_1 cytokines compared to uncomplicated cases [4,5], and increased immune cell accumulation and cytokine transcription in the brain [6,7]. These local and systemic responses are thought to contribute to CM by activating brain endothelium, leading to upregulated cell adhesion molecules, parasite sequestration, and microvascular obstruction [8]. Murine models of CM – which replicate many but not all aspects of human CM – lend causal support to these mechanisms [9-11]. In SMA, relative increases in T_H_1 cytokines [12,13] may contribute to anaemia by suppressing erythropoiesis and enhancing erythrophagocytosis [14,15]. At the molecular level, pattern recognition receptors (e.g., Toll-like receptors (TLRs) [16]) and cytokines (e.g., IFN-γ [17]) have been implicated in these inflammatory responses, but the pathways remain ill-defined.

Chitinase 3-like 1 (CHI3L1) is a 40 kDa secreted glycoprotein from the highly conserved 18 glycosyl hydrolase family. CHI3L1 is structurally similar to chitinases and can bind chitin, but lacks chitinase activity due to altered active site residues [18,19]. It is secreted by a variety of cell types, including macrophages, neutrophils, fibroblasts, astrocytes, and tumour cells [20]. Expression is induced by stimuli such as cytokines, radiation, and hypoxia [21-23]. CHI3L1 levels are increased in chronic inflammatory conditions and acute infections such as pneumonia, meningitis, and sepsis [24-26], and often correlate with disease severity and prognosis [20].

CHI3L1 has been implicated in diverse biological processes, including tissue remodelling, angiogenesis, and cell survival (reviewed in [20]). Of relevance to malaria, CHI3L1 has been shown to modulate immune responses. CHI3L1 contributed to induction and propagation of pathological T_H_2 responses in a murine asthma model. *Chi3l1*^*−/−*^ mice had decreased lung inflammation, secondary to increased apoptosis of immune cells [27]. Conversely, *Chi3l1* deletion worsened outcome in a murine model of *Streptococcus* pneumonia, with increased bacterial burden, lung inflammation, T_H_1 cytokines, and death. This was attributed to enhanced inflammasome activation and macrophage pyroptosis, leading to poor infection control [28]. Thus, CHI3L1 appears to skew immune responses towards a T_H_2 profile, and depending on underlying disease mechanisms, may promote or protect against immunopathology.

Given the association of severe malaria with excessive T_H_1 inflammatory responses, it is reasonable to hypothesize that CHI3L1 levels would be increased in severe malaria infection, and would protect against immunopathology in experimental CM (ECM). This report demonstrates that plasma CHI3L1 levels in Ugandan children presenting with *P. falciparum* malaria correlated with disease severity and predicted fatal outcome. Exposure of human immune cells to *P. falciparum* parasitized erythrocytes induced CHI3L1 expression, and plasma and brain CHI3L1 were increased in the *Plasmodium berghei* ANKA model of ECM. However, targeted *Chi3l1* deletion did not affect inflammatory parameters or survival in this model.

## Methods

### Study population and ethics statements

The study population has been previously described [29]. Briefly, this case–control study is nested within a prospective observational case–control study at Mulago Hospital in Kampala, Uganda [30]. In the larger study, paediatric patients (six months to 12 years old) presenting with fever and microscopy-confirmed *P. falciparum* infection were eligible for enrollment. Cases were defined as children who died and/or fulfilled World Health Organization criteria for CM, SMA, hypoxia, or lactic acidosis [31], while controls were age-matched children lacking these criteria (“uncomplicated malaria”, UM). Exclusion criteria included severe malnutrition, HIV co-infection, sickle cell trait/disease, absence of adequate consent, or death prior to collection of any laboratory specimens. After informed consent, clinical and demographic data and venous blood samples were collected. Citrate plasma was aliquoted and stored at −20°C until testing. Thin blood smears obtained at presentation were reviewed at a reference parasitology laboratory by two independent experts to determine parasite density. For the biomarker analysis, CM and SMA inpatients (cases) and UM outpatients (controls) were selected from the larger study based on availability of previously unthawed plasma. Ethical approval for the study was obtained from the Mulago Hospital Research Ethics Committee, Makerere University Faculty of Medicine Research Ethics Committee, Uganda National Council for Science & Technology, and the University Health Network. Written informed consent was obtained from parents/guardians before enrollment.

### CHI3L1 quantification

Plasma samples were assayed using the CHI3L1 Duoset ELISA kit (R&D Systems) according to the manufacturer’s instructions with the following changes: assays were performed in 50 μL/well; plasma samples were incubated overnight at 4°C; and ELISAs were developed using Extravidin®-Alkaline Phosphatase (Sigma, 1:1000, 45 min) followed by addition of p-Nitrophenyl phosphate substrate (Sigma) and optical density readings at 405 nm.

### *Plasmodium falciparum* culture

*Plasmodium falciparum* (ITG strain) was cultured as previously described [32]. Cultures were treated with Mycoplasma-Removal Agent (MP Biochemicals), confirmed to be mycoplasma-free (MycoAlert Mycoplasma Detection Kit, Lonza), and synchronized by alanine treatment [33]. Mature-stage cultures were used in assays.

### Peripheral blood mononuclear cell (PBMC) isolation and stimulation

Human PBMCs were isolated from venous blood of healthy volunteers by gradient centrifugation using Ficoll-Paque (GE Healthcare). PBMCs were suspended in RPMI 1640 with 10% foetal bovine serum and gentamicin (Gibco-Invitrogen) and incubated with medium alone, LPS (10 ng/mL), *P. falciparum* parasitized erythrocytes (3:1), or uninfected erythrocytes. Conditions were performed in duplicate or triplicate. Cell-free supernatants were collected and analysed by CHI3L1 ELISA (R&D Systems). Cells were gently scraped and cDNA was prepared as described below.

### Quantitation of human CHI3L1 mRNA

Total RNA was purified from PBMCs using the RNeasy kit (Qiagen). Following DNase treatment (Fermentas), mRNA was converted to cDNA (iScript, Bio-Rad Laboratories) and subjected to quantitative real-time PCR analysis for CHI3L1 mRNA levels (forward primer: 5’-TGCCCTTGACCGCTCCTCTGTACC-3’; reverse primer: 5’-GAGCGTCACATCATTCCACTC-3’; standard cycling protocol). Copy number was interpolated from a standard curve of genomic DNA and normalized to the geometric mean of housekeeping genes *Hbms*, *Ywhaz*, and *B2m*.

### Experimental cerebral malaria model

*Chi3l1*^−/−^ mice on a C57BL/6 background [27] were bred with wild-type C57BL/6 mice (The Jackson Laboratory, Maine, USA) to generate heterozygotes, which were then bred to produce *Chi3l1*^−/−^ mice and wild-type littermates. Frozen stocks of *P. berghei* (strain ANKA, MRA-311, MR4 ATCC, Virginia, USA) were passaged through C57BL/6 mice to obtain infected blood. Female mice (6–10 weeks old) were administered 1×10^6^ infected red blood cells by intraperitoneal injection. Tail vein blood smears were stained with Hema-3 Stain Set (Fisher Scientific) to determine parasitaemia. Mice were euthanized upon development of neurological signs, severe dehydration, or lethargy. Animal protocols were approved by the University of Toronto Animal Care Committee and all animal work was performed in compliance with university institutional guidelines.

### Plasma protein determination in ECM model

Mice were euthanized by inhaled CO_2_ at various times during infection, blood was obtained by cardiac puncture, and plasma was assayed by mouse CHI3L1 ELISA (R&D Systems). Saphenous vein blood was collected on Day 0 and 4 of infection and cytokines were measured in plasma using Cytometric Bead Array (Mouse Inflammation Kit, Becton Dickinson).

### Quantification of brain CHI3L1 transcripts in ECM model

*Plasmodium berghei* ANKA-infected mice were euthanized, brains were snap-frozen in liquid nitrogen and stored at −80°C. RNA extraction was performed as follows: samples were homogenized in Trizol (Invitrogen), briefly incubated at room temperature, and mixed with chloroform. The aqueous phase was collected and RNA was precipitated with isopropanol at room temperature. RNA pellets were washed with 75% ethanol, dried, and dissolved in water. DNase treatment, conversion to cDNA, and qRT-PCR were performed (CHI3L1 forward primer: 5’-GTACAAGCTGGTCTGCTACTTC-3’; Reverse primer: 5’-ATGTGCTAAGCATGTTGTCGC-3’). Copy number was interpolated from a genomic DNA standard curve and normalized to the geometric mean of *Gapdh*, *Ywhaz*, *Hrpt*.

### Statistical analysis

Analyses were performed using GraphPad Prism v4.03 (San Diego, CA), IBM SPSS v22.0 (Armonk, NY), and MedCalc Statistical Software v13.0.6 (Ostend, Belgium). Differences between groups were assessed using the Mann–Whitney U test or Kruskal-Wallis test with Dunn’s post-tests. Survival curves were compared using the log-rank test. Receiver operating characteristic (ROC) curves were generated and compared using the non-parametric method of Delong et. al. Biomarker cut-points were determined using the Youden index (J = max[sensitivity + specificity – 1]). CHI3L1 was combined with other biomarkers identified in a previous publication [29]: angiopoietin-2, CXCL10 (IP-10), procalcitonin, soluble ICAM-1, soluble FLT-1, and soluble TREM-1. Positive and negative predictive values were calculated using the reported case fatality rate of 5.7% for CM and SMA at Mulago Hospital [34].

## Results

### Plasma CHI3L1 is elevated in children with severe versus uncomplicated malaria

**Figure 1:**
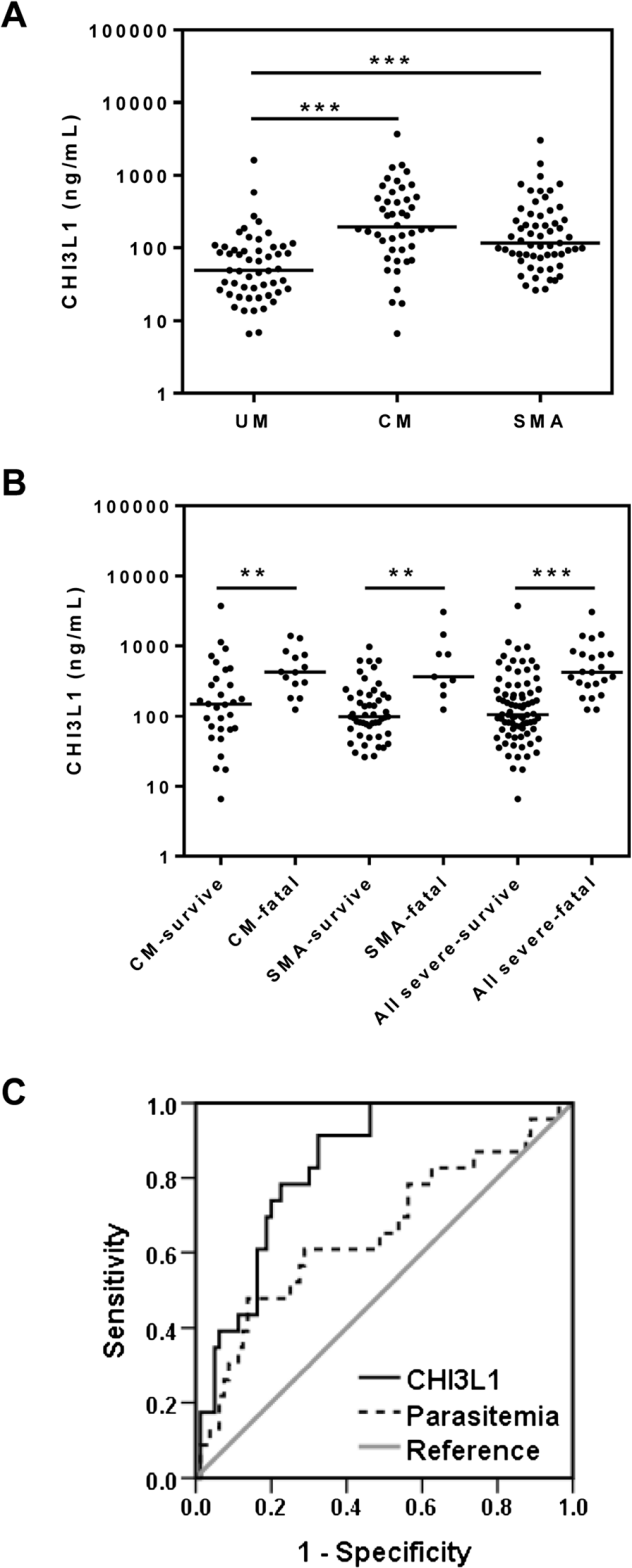
**Plasma CHI3L1 levels are increased in children with severe malaria and are predictive of outcome. (A)** CHI3L1 was measured by ELISA in the plasma of Ugandan children presenting to Mulago Hospital with uncomplicated malaria (UM, n = 53), cerebral malaria (CM, n = 44), and severe malarial anaemia (SMA, n = 59). **(B)** CM and SMA patients were analyzed based on the outcome of infection (i.e., survival versus fatality). CM and SMA were examined separately (left side), then pooled together (right side) for further analysis. Data are presented as dot plots with medians. **p < 0.01 and ***p < 0.001, Kruskal-Wallis test with Dunn’s post-hoc tests. **(C)** Receiver operating characteristic (ROC) curve analysis to assess the utility of CHI3L1 for predicting outcome of infection. Area under the curve for CHI3L1 is 0.84 (95% CI 0.76-0.92). Parasitaemia (often used clinically for prognosis) is shown for comparison with area under the curve 0.66 (95% CI 0.56-0.75).

To determine whether CHI3L1 correlates with disease severity in human malaria, plasma levels of CHI3L1 were compared among groups of Ugandan children presenting to hospital with *P. falciparum* infection: uncomplicated malaria (UM; n = 53), CM (n = 44), and SMA (n = 59) [29]. Demographic and clinical characteristics are shown in Table 1. Groups were comparable, except that children with SMA were younger with longer duration of illness prior to presentation and lower parasitaemia. At time of presentation to hospital, plasma CHI3L1 was significantly higher in children with CM and SMA compared to uncomplicated malaria (p < 0.001; Figure 1A), indicating that CHI3L1 levels reflect disease severity in malaria infection.

**Table 1:** **Demographic and clinical characteristics of study participants presenting with uncomplicated and severe malaria**^**a**^

**Characteristic**	**UM**^**b **^**(n = 53)**	**CM**^**c **^**(n = 44)**	**SMA (n = 59)**	**Pooled severe malaria**	
				**Survivors (n = 80)**	**Fatalities (n = 23)**
**Gender (% female)**	45.3	52.3	49.2	46.3	65.2^§§^
**Age (years)**	4.4 (2.1, 8.1)	3.0 (1.5, 4.3)	1.3 (0.9, 2.0)^***###^	1.6 (1.0, 3.1)	1.9 (1.2, 3.3)
**Days reported ill prior to presentation**	3 (2, 4)	3 (2, 4)	4 (3, 5)^***#^	3 (3, 4)	3 (2, 7)
**Parasitaemia (parasites/μL)**	3.8 × 10^4^ (1.6 × 10^4^, 1.2 × 10^5^)	9.8 × 10^4^ (1.5 × 10^4^, 2.7 × 10^5^)	2.6 × 10^4^ (7.4 × 10^3^, 1.2 × 10^5^)^#^	3.7 × 10^4^ (7.5 × 10^3^, 1.5 × 10^5^)	1.6 × 10^5^ (2.2 × 10^4^, 3.9 × 10^5^)^§^
**Fatal cases**	0	14	9	0	23

^a^Table reproduced from the previous study of this population (published in open access journal) [29]. Variables are presented as median (interquartile range). Groups were compared using the Mann Whitney U test or Kruskal-Wallis test with Dunn’s post-hoc tests (continuous variables) or Chi-square test (categorical variables). ^b^UM, uncomplicated malaria; CM, cerebral malaria; SMA, severe malaria anaemia. ^c^6 children with concurrent CM and SMA were included in the CM group. 5 children with SMA exhibited decreased consciousness but did not meet criteria for CM. ^***^p < 0.001 CM or SMA vs. UM. ^#^p < 0.05, ^###^p < 0.001 SMA vs. CM. ^§^p < 0.05, ^§§^p < 0.01 fatalities vs. survivors.

### CHI3L1 levels predict fatality in severe malaria

Next, it was assessed whether CHI3L1 could prognosticate outcome among children with severe malaria, which could facilitate clinical triage and resource allocation. CHI3L1 levels were higher among children with CM and SMA who subsequently went on to die of their infection, compared to survivors in their respective groups (p < 0.01; Figure 1B). As CHI3L1 values were similar between CM and SMA, and there is often clinical overlap between syndromes, all children with severe disease were combined, with similar results (Figure 1B; Table 1). This grouping was used for further analysis. CHI3L1 showed good discriminatory ability between fatalities and survivors in ROC curve analysis (Figure 1C; area under ROC curve [AUROCC] 0.84, 95% CI 0.76-0.92). This was comparable to the best-performing host biomarkers in previous reports [29,35,36]. CHI3L1 was also superior to parasitaemia (Figure 1C; AUROCC 0.66, 95% CI 0.56-0.75, difference between AUROCCs 0.181, 95% CI 0.037-0.33, p = 0.014), which is often used to evaluate severity in clinical settings.

To quantify the ability of CHI3L1 to predict outcome in severe malaria, a numerical cut-point was selected based on the Youden Index (see Methods Section). A cut-point of 179.1 ng/mL had high sensitivity (91.3%) but low specificity (67.5%) for predicting mortality (Table 2). To improve upon the accuracy of CHI3L1, CHI3L1 was combined with other biomarkers that were previously found to predict outcome in this sample [29]. For all possible two- or three-marker combinations involving CHI3L1, an additive scoring system was applied to each patient: one point was assigned for each biomarker value above its respective cut-point, and these were summed. Scores were evaluated for predictive ability. This analysis yielded combinations with >90% sensitivity and >80% specificity for predicting mortality, with negative predictive values >99% (Table 2). These results indicate that plasma CHI3L1, in combination with other biomarkers, may have clinical utility for prognosis in children with severe malaria.

**Table 2:** **Performance of CHI3L1 alone and in combination for predicting mortality among children with severe malaria**^**a**^

**Markers**	**Cut-point**^**b**^	**Sensitivity (%)**	**Specificity (%)**	**PLR**^**c**^	**NLR**	**PPV (%)**^**d**^	**NPV (%)**
CHI3L1	≥179.1 ng/mL	91.3 (73.2-97.6)	67.5 (56.6-76.7)	2.8 (2.0-3.9)	0.13 (0.034-0.49)	14.5 (5.0-30.3)	99.2 (93.1-100)
CHI3L1, IP-10, sICAM-1	≥2 points	91.3 (73.2-97.5)	83.8 (74.2-90.3)	5.6 (3.4-9.4)	0.10 (0.028-0.39)	25.4 (9.2-48.8)	99.4 (94.4-100)
CHI3L1, Ang-2, IP-10	≥2 points	100 (85.7-100)	81.3 (71.3-88.3)	5.3 (3.4-8.4)	--	24.4 (9.4-46.0)	100 (95.4-100)
CHI3L1, Ang-2, sICAM-1	≥2 points	95.7 (79.0-99.2)	81.3 (71.3-88.3)	5.1 (3.2-8.1)	0.054 (0.008-0.37)	23.6 (8.6-45.7)	99.7 (94.8-100)

^a^All parameters are presented with 95% CIs in parentheses. ^b^Cut-points were determined using the Youden Index (J = max[sensitivity + specificity – 1]). ^c^PLR, positive likelihood ratio; NLR, negative likelihood ratio; PPV, positive predictive value; NPV, negative predictive value; sICAM-1, soluble ICAM-1, Ang-2, angiopoietin-2. ^d^PPVs and NPVs were based on estimates that 5.7% of CM and SMA patients at Mulago hospital die of their malaria infection [34].

### *Plasmodium falciparum* stimulates CHI3L1 production by human immune cells

**Figure 2:**
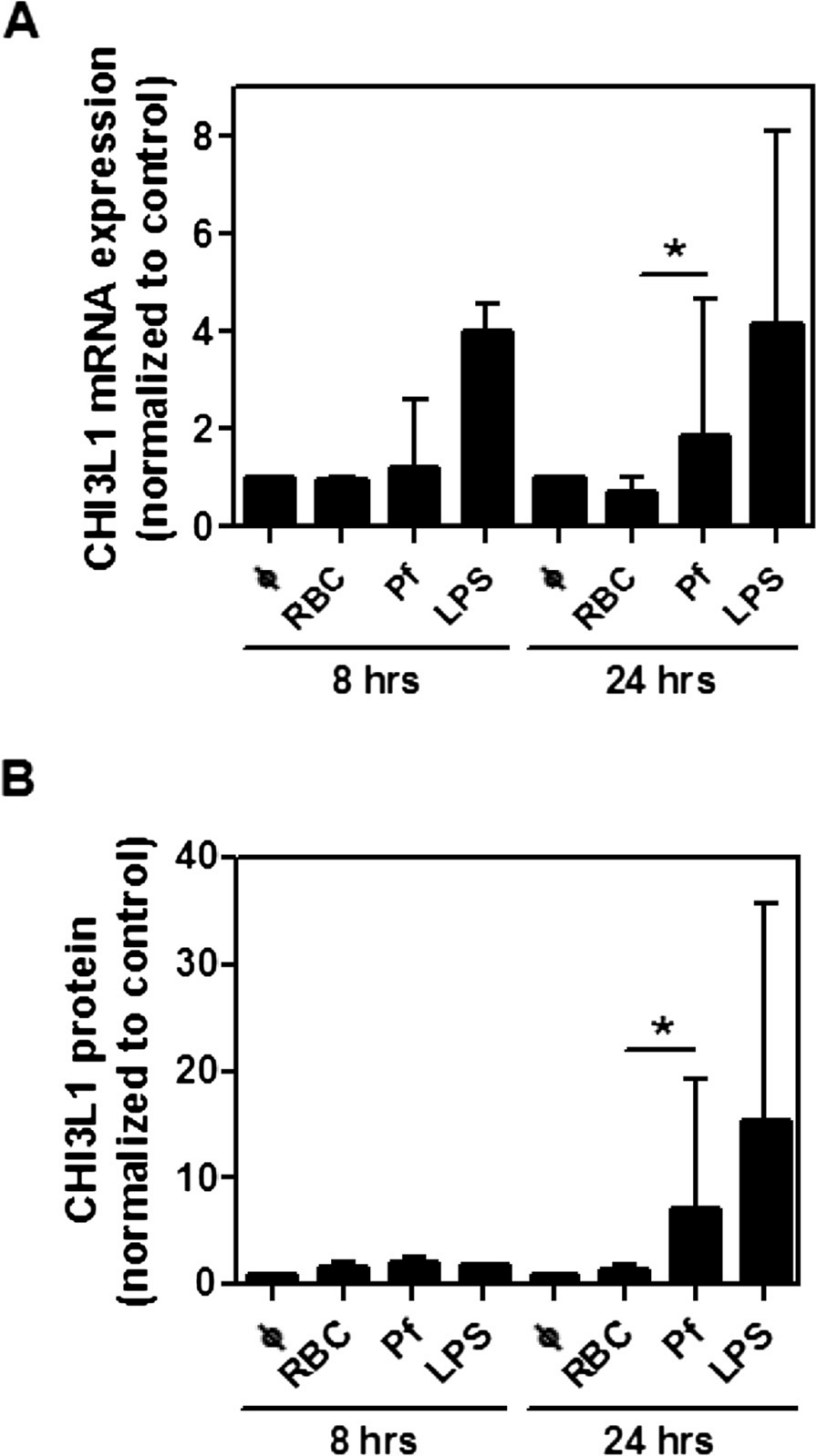
**Human PBMCs produce CHI3L1 upon stimulation with *P. falciparum*.** PBMCs were exposed to medium alone, uninfected red blood cells (RBCs; negative control), *P. falciparum* infected RBCs (Pf), or LPS (positive control) for 8 and 24 hrs. **(A)***Chi3l1* mRNA was assessed by quantitative real-time PCR, and **(B)** CHI3L1 concentration in supernatants was measured by ELISA. Graphs represent pooled data from at least 3 independent experiments using different PBMC donors. Data are presented as medians with interquartile ranges. *p < 0.05, Kruskal-Wallis test with Dunn’s post-tests.

Next, potential sources of CHI3L1 during malaria infection were examined. As malaria is a blood-borne infection, *P. falciparum*-infected erythrocytes were co-cultured with human peripheral blood mononuclear cells (PBMCs). As a positive control, LPS stimulated CHI3L1 transcription (Figure 2A) and protein secretion (Figure 2B) [37]. Compared to incubation with uninfected red blood cells, exposure to *P. falciparum-*infected erythrocytes for 24 hours induced an average 2-fold increase in CHI3L1 transcription (Figure 2A), and also significantly increased secretion of CHI3L1 protein (Figure 2B).

### CHI3L1 levels are increased in experimental cerebral malaria, but outcome is unaffected by *Chi3l1* deletion

The regulation and role of CHI3L1 in vivo were then investigated using a murine model of CM. Infection of C57BL/6 mice with *P. berghei* strain ANKA produces a neurological syndrome similar to CM, characterized by paralysis, ataxia, convulsions, and death between days 6–12 [11]. This model recapitulates several features of brain histopathology in human CM, including ring haemorrhages, activated endothelium with vascular leak, and monocyte and parasite accumulation [38,39]. There are some differences between human CM and ECM: in ECM, there is greater immune cell sequestration and cerebral oedema, and less PE sequestration. However, having established a clinically relevant role for CHI3L1 as a marker of disease severity and death in human severe malaria, this model provides a rational approach to examine mechanism and causality of this pathway in the pathobiology of severe disease that would be problematic to investigate directly in humans.

**Figure 3:**
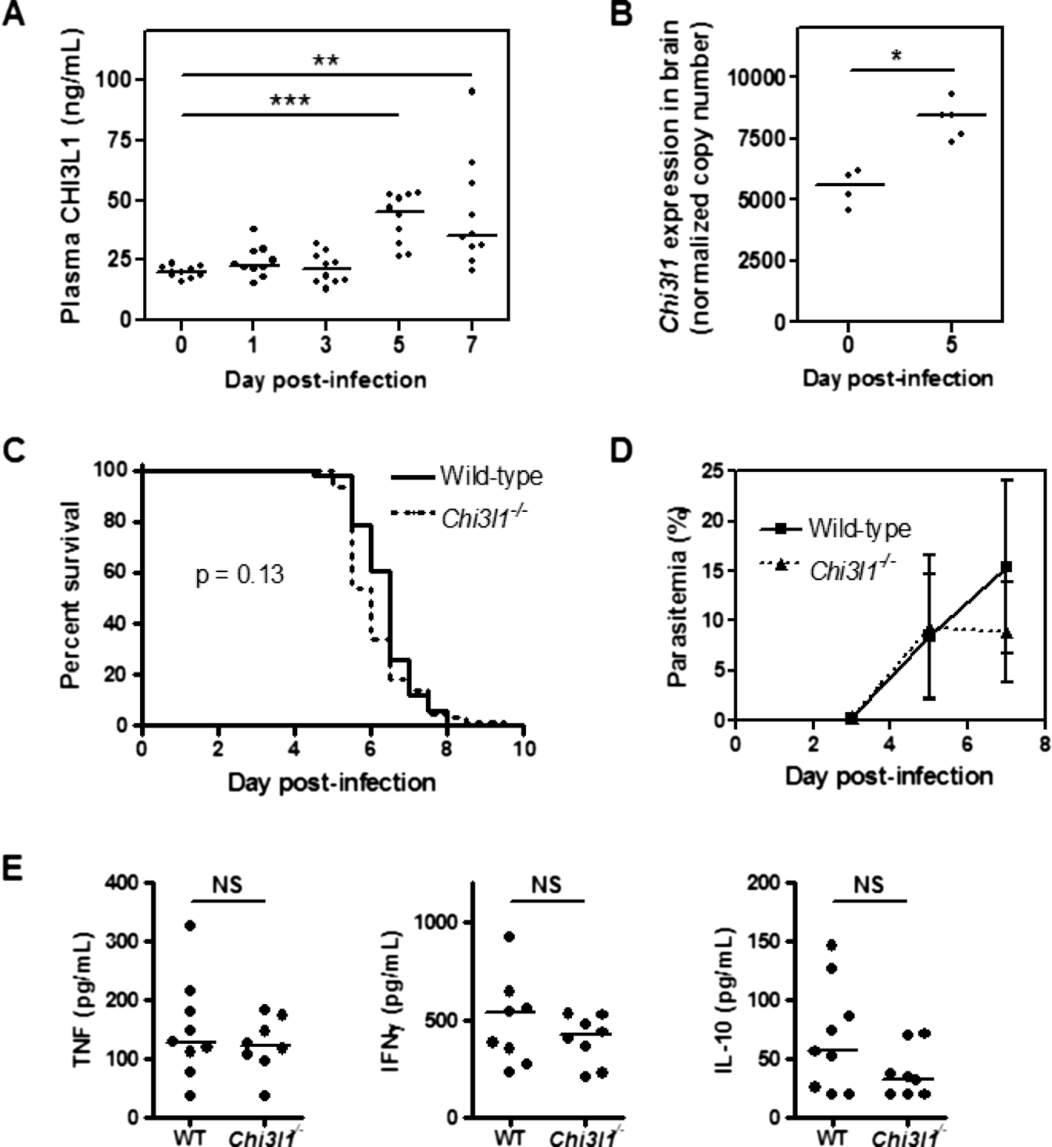
**CHI3L1 increases during experimental cerebral malaria but does not affect survival. (A)** Plasma was collected over a time course for *P. berghei* ANKA infection of C57BL/6 mice, and assayed for CHI3L1 by ELISA. **p < 0.01, ***p < 0.001 by Kruskal-Wallis test with Dunn’s post-test. **(B)** On Days 0 and 5 of infection, *Chi3l1* transcripts in brain were measured by quantitative real-time PCR and normalized to housekeeping genes. *p < 0.05 by Mann–Whitney U test. **(C)** Four independent experiments were pooled to generate the survival curves shown (*Chi3l1*^−/−^, n = 65; Wild-type littermates (WT), n = 51). The log-rank test was used to assess survival differences (p = 0.13). **(D)** Parasitaemia is represented as medians and interquartile range. **(E)** Plasma was collected from wild-type and *Chi3l1*^−/−^ mice on Day 4 of infection and assayed for cytokines. NS, not significant, Mann Whitney U test. Cytokines were undetectable in all uninfected mice.

Plasma CHI3L1 was increased in mice on Days 5 and 7 following *P. berghei* infection (Figure 3A). Using quantitative real-time PCR, *Chi3l1* mRNA expression in the brain was significantly elevated on Day 5 compared to uninfected mice (Figure 3B). To assess whether these elevations in CHI3L1 correspond to a functional role in ECM, *Chi3l1*^−/−^ mice and their wild-type littermate controls were infected with *P. berghei*. There were no differences between wild-type mice and *Chi3l1*^−/−^ mice in terms of survival (Figure 3C; p = 0.13 by log-rank test), parasitaemia (Figure 3D), or plasma levels of pro-inflammatory T_H_1 (TNF, IFN-γ) or anti-inflammatory (IL-10) cytokines (Figure 3E). Thus, while local and systemic CHI3L1 levels increase in ECM-susceptible mice during *P. berghei* infection, CHI3L1 does not appear to affect immune responses or outcome in this model.

## Discussion

This study represents the first investigation into the regulation and role of CHI3L1 in malaria infection. Plasma CHI3L1 was elevated in Ugandan children with CM and SMA compared to uncomplicated malaria. Moreover, CHI3L1 levels at presentation predicted mortality in severe disease, particularly when combined with other host biomarkers. These data indicate that CHI3L1 may have clinical utility as a prognostic test component. Using model systems, it was found that malaria parasites stimulated CHI3L1 production by human immune cells *in vitro*, and plasma and brain CHI3L1 increased during ECM. However, ECM-susceptible mice and *Chi3l1*^−/−^ littermates did not show differences in terms of inflammatory cytokines or survival, suggesting that CHI3L1 does not critically contribute to ECM pathogenesis in this model.

Consistent with the original hypothesis, plasma CHI3L1 levels were associated with disease severity in malaria infection. Inflammation is a known inducer of CHI3L1, and a well-established feature of severe malaria. Although CM and SMA are often considered to have distinct pathophysiological mechanisms, excessive T_H_1 inflammatory responses are common to both syndromes [4,5,12,13]. Another trigger for CHI3L1 upregulation is hypoxia, which may also be shared between CM and SMA, albeit for different reasons (microvascular obstruction and decreased oxygen carrying capacity, respectively). Thus, it is reasonable that CHI3L1 would be similarly elevated in both syndromes, and further increased in children with pathology severe enough to cause death.

CHI3L1 alone had excellent sensitivity (>90%) but poor specificity (<70%) for predicting mortality in children with severe malaria. Combining CHI3L1 with other host biomarkers improved predictive accuracy, which has important practical implications in resource-poor health care settings with limited availability of intensive care. Severe malaria patients at higher risk of death could be prioritized for referral and more intensive monitoring and supportive care – although further studies would be required to determine whether these measures could improve outcome. Moreover, in clinical trials of adjunctive therapies for severe malaria, a prognostic test could be used to stratify patients into risk categories, since treatment efficacy can vary depending on risk of death [40]. While clinical and laboratory parameters have been associated with mortality in severe malaria [41-43], predictive accuracy is not ideal. A quantitative point-of-care biomarker panel based on emerging technologies [44] could enable rapid, objective assessment.

Prospective studies in multiple populations are required to confirm the findings in this report. The current study had some limitations, including small sample size and non-consecutive sampling, possibly introducing selection bias. Future studies should also evaluate whether common childhood co-infections associated with elevated CHI3L1, such as pneumonia and schistosomiasis [26,45], affect the accuracy of CHI3L1 as a prognostic indicator in malaria infection. CHI3L1 elevation alone is likely too non-specific to identify retinopathy-positive CM, which approximates “true” CM [46,47]. However, the prognostic ability of CHI3L1 in combination with other host biomarkers should be evaluated in this group, particularly as surrogate markers for retinopathy are in development for use in clinical settings [35,48].

Increased plasma CHI3L1 in severe and fatal human malaria prompted examination of CHI3L1 in malaria models. *Plasmodium falciparum *stimulated CHI3L1 production by human PBMCs in vitro. Activated monocytes were likely the main source of CHI3L1 in this system; while CHI3L1 is not expressed by freshly isolated monocytes, differentiation into macrophages – as might be triggered by interaction with *P. falciparum* – induces its transcription [49]. Additional cell types may have contributed to increased plasma CHI3L1 in *P. berghei* infection, such as neutrophils, which are activated during malaria infection [50,51] but excluded from PBMC preparations. CHI3L1 expression in the brain was also increased during *P. berghei* infection in mice susceptible to ECM, potentially due to upregulated transcription in astrocytes [23,52] or intravascular monocyte sequestration [11].

The precise pathways mediating CHI3L1 induction by malaria remain undefined. Pro-inflammatory cytokines such as TNF and IL-1β can stimulate CHI3L1 production [21,22]. *Plasmodium falciparum* components have been shown to induce these cytokines via activation of pattern recognition receptors such as TLRs [53-55]. Notably, TLR2 and TLR4 agonists induce CHI3L1 in monocytic cell lines [37,56], though it is unclear whether direct TLR signalling or the resultant cytokine secretion mediates the effect. To address this question, cycloheximide was added to the co-culture system, but this agent has anti-malarial activity and abolished PBMC responses (data not shown). Of note, hypoxia stimulated CHI3L1 production in a glioblastoma cell line [23], which promotes cell survival [57]. This suggests that CHI3L1 upregulation in the brain in ECM in response to hypoxia and/or cytokines could be a neuroprotective mechanism.

However, in this study, there was no global effect of *Chi3l1* deletion on *P. berghei* infection of ECM-susceptible mice. It was initially hypothesized that CHI3L1 would decrease pathological T_H_1 inflammation in ECM, and that, similar to the *Streptococcal* pneumonia model, *Chi3l1* deletion would worsen outcome. In fact, *Chi3l1* deletion failed to alter measures of T_H_1 inflammation in ECM. This may be due to differential pathway engagement: CHI3L1 exerted its major effects in the pneumonia model by regulating the NRLP3 inflammasome [28], while involvement of the classical NRLP3 inflammasome in ECM pathogenesis is doubtful [58,59]. Alternatively, any worsening of outcome may have been counterbalanced by beneficial effects of *Chi3l1* deletion. CHI3L1 could theoretically promote pathological inflammatory responses by supporting survival of immune cells [27] or activating endothelial cells [60]. In the ECM model, CD8+ T cells and monocytes accumulate in brain microvasculature [39,61], where they secrete mediators such as perforin that directly cause endothelial injury [62] and cytokines that promote endothelial activation, parasite sequestration, and vascular leak [10,38,63]. Multiple, competing roles for CHI3L1 have also been observed in a model of cigarette smoke exposure, in which CHI3L1 promoted pathological inflammatory infiltrates yet also protected lung epithelial cells from apoptosis [64].

Finally, it may simply be technically challenging to detect worsened outcome in ECM-susceptible mice. There are scant examples in the literature of single gene alterations leading to earlier death in this model, and these have been associated with more rapid parasite replication [65]. It may be that a critical parasite burden is required to initiate the central pathological pathways of ECM, and the subsequent decline is so time-compressed that it is difficult to discern earlier onset of morbidity or mortality.

## Conclusions

In Ugandan children with malaria, plasma levels of CHI3L1 were elevated in severe disease and further increased in those who subsequently died of infection. In combination with other host biomarkers, CHI3L1 holds promise as a prognostic biomarker in severe malaria infection; further studies are warranted to validate this finding. Although deletion of *Chi3l1* did not alter key disease parameters or death in the *P. berghei* model of ECM, these findings do not exclude a role for CHI3L1 in human severe malaria because of model limitations and, potentially, the multiplicity of CHI3L1 functions.

## Abbreviations

AUROCC: Area under receiver operating characteristic curve; CHI3L1: Chitinase 3-like 1; CM: Cerebral malaria; ECM: Experimental cerebral malaria; PBMC: Peripheral blood mononuclear cells; RBC: Red blood cell; ROC: Receiver operating characteristic; SMA: Severe malarial anaemia; TLR: Toll-like receptor; UM: Uncomplicated malaria.

## Competing interests

LKE, WCL, and KCK are named inventors on a patent owned by the University Health Network covering biomarkers for early determination of critical response to illness and treatment.

## Authors’ contributions

LKE conceived of the study, carried out experiments, and drafted the manuscript. CP contributed to study design and in vitro and in vivo studies. ZL carried out in vivo studies. AD, CM, and CMC-G collected human plasma samples and critically revised the manuscript. CGL and JAE generated knockout mice and critically revised the manuscript. WCL and KCK conceived of the study and helped draft the manuscript. All authors read and approved the final manuscript.
